# Ramadan fasting alters food patterns, dietary diversity and body weight among Ghanaian adolescents

**DOI:** 10.1186/s12937-018-0386-2

**Published:** 2018-08-11

**Authors:** Zakari Ali, Abdul-Razak Abizari

**Affiliations:** Department of Nutritional Sciences, School of Allied Health Sciences, University for Development Studies, P O Box 1883, Tamale, Ghana

**Keywords:** Ramadan, Fasting, Adolescents, Dietary diversity, Weight change, Ghana

## Abstract

**Background:**

Ramadan is a monthlong fast for healthy adolescents and adult Muslims. The quality of foods eaten and eating patterns in Ramadan may be different from other months of the year. Food intake of adolescents is a concern as energy and nutrient requirements are higher and needed to support the growth spurt of this stage. The objective of the present study was to describe the food patterns, dietary diversity and body weight changes among adolescents during Ramadan.

**Methods:**

A prospective cohort study design with four measurement points (baseline, midline, endline and post endline) was conducted among 366 adolescents in Junior High Schools. Food pattern was assessed with a food frequency questionnaire, a 24-h dietary recall was used to assess dietary diversity and body weight was measured using an electronic scale. A repeated measures ANOVA was used to compare changes in dietary diversity scores (DDS) and weight of pupils.

**Results:**

Half of the pupils (50.3%) were female and average age was 15.9 ± 1.8 years. Pupils fasted for an average of 28.3 ± 4.0 days and 14.3 ± 0.5 h a day (dawn to dusk) during Ramadan. The number and types of dishes taken at meal times differed substantially between Ramadan periods and outside Ramadan. Consumption of vitamin A-rich fruits, other fruits, and milk and milk products increased markedly during Ramadan. However, fasting came with a reduction in consumption of foods from roots and tubers, legumes and nuts, and dark green leafy vegetables while other food groups remained unchanged. Mean DDS increased significantly during Ramadan (F (2.933, 1070.573) = 7.152, *p* < 0.001) while mean daily meal frequency decreased (F (2.936, 1071.623) = 51.653, *p* < 0.001). There was significant body weight loss (-1.5 kg (95% CI: -1.1 kg to -1.6 kg)) among adolescents (F (2.656, 958.95) = 304.90, *p* < 0.001). Weight loss was short-lived; regained one month after Ramadan.

**Conclusion:**

In this prospective cohort study among schooling Ghanaian adolescents who fast during Ramadan, fasting was characterised by marked changes in usual food patterns, increased dietary diversity and significant body weight loss.

**Electronic supplementary material:**

The online version of this article (10.1186/s12937-018-0386-2) contains supplementary material, which is available to authorized users.

## Background

Ramadan is the ninth month of the Islamic lunar calendar and healthy adolescent and adult Muslims refrain from eating or drinking from dawn to dusk for 29 or 30 days [1]. During Ramadan, Muslims fast for an average period of 12 h during the day with a common practice of consuming one large meal after dusk and a lighter meal before dawn [2]. Although children who have not reached puberty are exempt from fasting [3], it is common to find some who practice fasting at 6 or 8 years of age [4] and by early adolescence many children participate fully in Ramadan fasting [5]. Meals taken during the day are normally reduced to two [6] and often little time is available for more meals before bedtime [4]. The quality of foods eaten and eating patterns are changed considerably [7]. The type of food eaten during the night in Ramadan may also be different from that usually consumed during the rest of the year [8]. Alterations in eating modes and infrequent meal schedules during Ramadan that lead to reduced food intake may affect important enzymatic and metabolic responses [9] and different aspects of human health [10]. The effect of fasting on human health has been the subject of a number of scientific investigations [2, 11]. However, mixed findings have been reported by studies on Ramadan fasting and health [12]. For instance, there have been mixed findings regarding body weight and important nutrient changes in response to Ramadan fasting [2]. There is, however, a general opinion that fasting has a potential non-pharmacological intervention for improving health and increasing longevity [2].

Adolescents have been reported to indulge in dietary behaviours that do not meet daily dietary recommendations [13] and a possible trigger of eating disorders [14] during Ramadan. This is a concern, as adolescence is a time of growth and development [15] and nutrient needs are higher than other periods of the life cycle [16]. Adequate nutrients and energy intake therefore, is critical to healthy growth and development at this stage of life [16].

Dietary diversity has proved to be an appropriate method to evaluate nutrient intake adequacy of individuals, including adolescents [17–19] and reflect specific nutrient adequacy [20].

There is, however, scarcity of data on the dietary diversity, food patterns and body weight changes of adolescents who fast during Ramadan. Present study, therefore aimed to fill this knowledge gap using adolescents in Junior High Schools in Northern Ghana, where majority of the people are Muslims.

## Methods

### Study area

The study was conducted in the Tamale Metropolis, one of the 26 districts of the Northern Region of Ghana. The Metropolis has a total estimated land size of 646.902 sqkm and located in the central part of the Region. The Metropolis has an estimated population of 233,252, comprising 49.7% males and 50.3% females with about 80.8% living in urban localities [21]. The Tamale Metropolis is dominated by Muslims; about 90.5% of the people practice Islamic religion. The population of the metropolis is youthful with about 36.4% aged below 15 years. About 60,000 of the children older than 3 years are enrolled in Primary Schools while 26,936 are enrolled in Junior High Schools (JHS) [22].

### Study design

A prospective cohort study design was used in the present study with four measurement points: baseline (before Ramadan), two times during Ramadan (midline and end line) and one month after Ramadan. The 2017 Ramadan fast started on 27th of May for the majority of the people and ended on 24th or 25th of June, lasting for 29 or 30 days. The baseline survey was conducted one week before the start of fasting. Midline survey was conducted at the end of the second week of fast while the end line survey was conducted in the last week (fourth week) of fasting. The final follow up (post Ramadan survey) was conducted one month after the end of fasting.

### Target population and sampling

The target population was adolescents aged 10 to 19 years enrolled in Junior High Schools within the Tamale metropolis. The Tamale Metropolis has 15 educational circuits with a total of 72 junior high schools. Half (7) of the circuits were selected using simple random sampling technique. The schools from the selected circuits were pooled together into a frame from which ten Junior High Schools (Anbariya Metropolitan Assembly (M/A) JHS Block ‘A’, Manhalia Islamic JHS, Datoyili T. I Ahmadiya JHS, Anbariya M/A JHS Block ‘B’, Lamashegu M/A JHS, Umar Al- Mukhtar JHS, Nahdah Islamic JHS, Zogbeli M/A JHS Block ‘A’, Kaladan Evangelical Presbyterian JHS and Wataniya Islamic JHS) were then selected by simple random sampling to form the participating schools. A total of 400 pupils in JHS 1 and 2 were then selected from the 10 schools using probability proportional to size (PPS) methodology. Pupils in their final year (JHS 3) were excluded from the study because they were falling out of follow up. Lists of pupils in JHS 1 and 2 of selected schools were compiled for each school using class registers to form the sampling frame. The required number of pupils for each school was selected from the sampling frame by simple random sampling technique using Excel generated random numbers.

### Data collection

Data collection was done using a pretested semi-structured questionnaire through face-to-face interviews. The questionnaire elicited information on pupil, parental and household sociodemographic characteristics, fasting and dietary intake characteristics. A similar questionnaire was used for all the follow-up surveys. Data collection staff were first degree nutritionists and received training on questionnaire administration and anthropometric measurements before each assessment. Data collection field supervisors also provided on-site questionnaire checks for completeness and all wrong responses and measurements were repeated and corrected the same day.

#### Assessment of dietary diversity and dish variety scores

A qualitative 24-h dietary recall was used for dietary intake assessment of pupils. Pupils were asked to recall all foods they consumed at home and outside the home (including at school) the day preceding the survey. Foods recalled were classified into their respective food groups based on which the dietary diversity was determined. The Food and Agriculture Organization (FAO) defines dietary diversity as the number of food groups an individual consumes over the past 24-h [23]. The dietary diversity of participants was assessed following standard guidelines for measuring individual dietary diversity by the FAO [23]. Dietary diversity score was calculated based on consumption of fourteen food groups including: Cereals; white roots and tubers; vitamin a rich vegetables and tubers; dark green leafy vegetables; other vegetables; vitamin a rich fruits; other fruits; organ meat; flesh meats, eggs; fish and seafood; legumes, nuts and seeds; milk and milk products; and oils and fats. The dietary diversity score therefore ranged from 0 to 14, a score of 1 was assigned if one food group was consumed and 14 if all food groups were consumed. The dish variety score was calculated as the number of dishes consumed in an eating moment. Composite dishes such as *Waakye* (mixture of rice and beans) with stew and porridge with bread were given single scores. A pupil therefore had a dish variety score of 2 if they had both *Waakye* with stew and porridge with bread at an eating moment (For example: breakfast). The dish variety scores were then categorized into ‘1’ when a pupil had a dish variety score of 1 and ‘2’ if they had a score of 2 or more at an eating moment to give the dish variety of that eating moment.

#### Assessment of dietary patterns

Dietary pattern was assessed using a food frequency questionnaire. The seven day food frequency questionnaire included 60 foods commonly consumed in northern Ghana and was similar to the one used earlier in the same area [24]. Pupils were asked to recall how often they have on average had a particular food in the past one week preceding each assessment. The consumption scores ranged from 0 (when they had never or hardly ever taken a particular food for the past week) to 7 (if they had a particular food for more than six days in the past one week). Changes in dietary patterns were assessed using changes in food consumption frequency during Ramadan relative to the non-Ramadan periods.

#### Weight and height measurements

Weight and height measurements were taken following WHO standard anthropometry guidelines [25]. Electronic weighing scales (Seca 874) were used for all weight measurements. Weights were recorded to the nearest 0.1 kg. The scales were regularly checked for accuracy and precision using a standard weight. Data collection staff were also trained to minimize measurement errors. Weight measurements were taken around the same time of the day for all pupils and in all schools for the four surveys.

### Data and statistical analysis

Data was entered, cleaned and analyzed using Statistical Package for Social Sciences (SPSS) for windows version 20. About 8.5% of the pupils had incomplete follow-up data. The a priori decision was to include only pupils who were available and completed all the four surveys. The analyses are therefore based on the remaining 91.5% (366) who completed all follow ups.

The data are presented as descriptive statistics in the form of frequencies and percentages for categorical variables and means and standard deviations for continuous variables. The association between mean dietary diversity scores, mean weight and sex of pupils at each assessment point was investigated using the independent student t-test. A repeated measures ANOVA was used to compare the changes in dietary diversity scores, daily meal frequency, and weight of pupils across the assessments. Differences were considered statistically significant at *p* < 0.05.

## Results

### Background characteristics of participating pupils in junior high schools in Tamale

Half of the pupils (50.3%) were female and average age was 15.9 ± 1.8 years. More than five in ten pupils (51.4%) were in JHS 1, belonged to the Dagomba ethnic group (87.2%) and were normal by BMI-for-age classification (87.7%). More than three-quarters of the pupils lived with their parents and almost all (92.6%) were given pocket money to school.

Though educational level of parents of pupils was generally low, 16.6% more of mothers had no formal education than fathers. While fathers (32.8%) mostly engaged in farming, the majority of mothers were traders (71.6%). Majority of fathers had more than one wife (57.4%) and mothers of participating pupils were the first wives (75.7%) of the fathers. Majority of pupils lived with extended families (58.5%) which were headed by their father (59.0%). More than six in ten (65.6%) of the pupils belonged to families of at least ten members (Table 1).

**Table 1:** Background characteristics of participating pupils in JHS

Characteristic	Summary values (*n* = 366)
Pupil characteristics	
Sex, n (%)	
Female	184 (50.3)
Age in years, (mean ± SD)	15.9 ± 1.8
Class of pupil, n (%)	
JHS 1	188 (51.4)
JHS 2	178 (48.6)
Ethnicity, n (%)	
Dagomba	319 (87.2)
Gonja	26 (7.1)
Others	21 (5.7)
Nutritional status (BMI-for-age classification), n (%)	
Underweight	23 (6.3)
Normal	321 (87.7)
Overweight/obese	22 (6.0)
Live with parents, n (%)	
Yes	286 (78.1)
Pocket money to school	
Yes	339 (92.6)
Parent characteristics	
Father’s educational level, n (%)	
None	195 (53.3)
Primary/JHS	98 (26.8)
SHS/Tertiary	73 (19.9)
Father’s occupation, n (%)	
Farmer	120 (32.8)
Trader	74 (20.2)
Civil servant	33 (9.0)
Others	139 (38.0)
Wives of father, n (%)	
At least two wives	210 (57.4)
Mother’s educational level, n (%)	
None	256 (69.9)
Primary/JHS	83 (22.7)
SHS/Tertiary	27 (7.4)
Mother’s occupation, n (%)	
Farmer	34 (9.3)
Trader	262 (71.6)
Civil servant	6 (1.6)
Others	64 (17.5)
Mother’s position, n (%)	
First wife	277 (75.7)
Other	89 (24.3)
Household characteristics	
Family type, n (%)	
Extended	214 (58.5)
Nuclear	152 (41.5)
Family head, n (%)	
Father	216 (59.0)
Grandfather	61 (16.7)
Mother	15 (4.1)
Grandmother	24 (6.6)
Others	50 (13.7)
Household size, n (%)	
At least 10 members	240 (65.6)
Number of household members below 20 years, mean ± SD	6.2 ± 4.1

### Fasting characteristics of pupils in participating junior high schools

The average age pupils started fasting was 11.2 ± 2.5 years. The pupils endured fasting for almost the whole month of Ramadan (28.3 ± 3.6 days) for an average of 14.3 ± 0.54 h each day (dawn to dusk). Almost all parents (98.1%) were also fasting and provided encouragement (80.34%) to their children; fathers (40.5%) encouraged pupils a little more than mothers (39.9%) to fast. Pupils fasted for a number of reasons including: spiritual growth (43.1%), compulsion by family (24.0%), parents fasting (8.1%) and for good health (3.0%).

Most pupils missed fast mainly due to menstruation (49.5%) and sickness (40.0%). Some pupils (39.9%) also fasted some number of days (5.4 ± 1.6) after Ramadan. Most pupils (63.1%) did not experience any sickness during fasting. However, those who reported some sickness mainly experienced diarrhoea (25.9%) and malaria (22.2%). Foods used for fasting were mainly obtained from home (64.5% at midline and 66.4% at endline). While foods obtained as gifts increased by about 2% between midline and endline assessments, bought foods decreased by nearly 4% between midline and endline assessments (Table 2).

**Table 2:** Fasting characteristics of participating pupils in JHS

Characteristic	Summary values
Age started fasting (*n* = 366), mean ± SD	11.2 ± 2.5^a^
Days fasted during Ramadan (*n* = 366), mean ± SD	28.3 ± 3.6^d^
Fasting duration within a day (hours) (*n* = 366), mean ± SD	14.3 ± 0.54^b^
Days missed fasting (mean ± SD)	1.73 ± 3.59^d^
Reasons for missing fasting, n (%)	
Could not wake up in good time	7 (6.7)^c^
Was sick	42 (40.0)^c^
Menstruation (girls)	52 (49.5)^c^
Others	4 (3.8)^c^
Parents fasting? (*n* = 363), n (%)	
Yes	356 (98.1)
Encouragement to fast (*n* = 351), n (%)	
Father	142 (40.5)^b^
Mother	140 (39.9)^b^
Sibling	15 (4.3)^b^
Uncle	13 (3.7)^b^
Spiritual leader	3 (0.9)^b^
Others	38 (10.8)^b^
Reasons for fasting (*n* = 334), n (%)	
Spiritual growth (blessing)	144 (43.1)^b^
It is compulsory	80 (24.0)^b^
Parents fasting	27 (8.1)^b^
For good health	10 (3.0)^b^
Other reasons	73 (21.9)^b^
Continued fasting after Ramadan (*n* = 366), n (%)	
No	220 (60.1)^d^
Days fasted after Ramadan (*n* = 146), mean ± SD	5.38 ± 1.55^d^
Reasons for fasting after Ramadan (*n* = 146), n (%)	
Obeying religious recommendation	83 (56.8)^d^
Replacing missed days	52 (35.6)^d^
Others	11 (7.5)^d^
Sickness during Ramadan? (*n* = 366), n (%)	
No	231 (63.1)^c^
Type of sickness during Ramadan (*n* = 135), n (%)	
Malaria	30 (22.2)^c^
Diarrhoea	35 (25.9)^c^
Others	70 (51.9)^c^
Source of midline fasting food (*n* = 366), n (%)	
Gifts	35 (9.6)
Purchase	87 (23.8)
Home	236 (64.5)
others	8 (2.2)
Source of end line fasting food (*n* = 366), n (%)	
Gifts	42 (11.5)
Buying	74 (20.2)
Home	243 (66.4)
Others	7 (1.9)

^a^measured at baseline ^b^measured at midline ^c^measured at endline ^d^measured at post Ramadan

### Changes in daily meal frequency among participating pupils in junior high schools during fasting

A repeated measure ANOVA with a Greenhouse-Geisser correction showed that the mean feeding frequency changed significantly across the assessment stages (F (2.936, 1071.623) = 51.653, *p* < 0.001). Post hoc test using Bonferroni correction revealed that mean feeding frequency decreased significantly between baseline and midline assessments (3.1 vs 2.7, *p* < 0.001) and between baseline and endline assessments (3.1 vs 2.7, *p* < 0.001) but not between baseline and post Ramadan (3.1 vs 3.1, *p* > 0.05) (Table 3).

**Table 3:** Pairwise comparisons of changes in feeding frequency of participating pupils in JHS before, during and aftrer Ramadan (Repeated measures ANOVA)

(I) feed frequency (mean ± SD)	(J) feed frequency (mean ± SD)	Mean Difference (I-J)	Std. Error	Sig.^b^	95% Confidence Interval for Difference^b^	
					Lower Bound	Upper Bound
Baseline frequency (3.1 ± 0.6)	Midline frequency (2.7 ± 0.8)	0.437^a^	0.048	< 0.001	0.311	0.564
	End line frequency (2.7 ± 0.7)	0.423^a^	0.044	< 0.001	0.308	0.539
	Post Ramadan frequency (3.1 ± 0.7)	0.052	0.042	1.000	−0.061	0.164
Midline frequency (2.7 ± 0.8)	End line frequency (2.7 ± 0.7)	− 0.014	0.048	1.000	−0.140	00.113
	Post Ramadan frequency (3.1 ± 0.7)	−0.385^a^	0.048	< 0.001	−0.513	−0.257
End line frequency (2.7 ± 0.7)	Post Ramadan frequency (3.1 ± 0.7)	−0.372^a^	0.047	< 0.001	−0.496	− 0.247

Based on estimated marginal means, ^a^. The mean difference is significant at the .05 level, ^b^Adjustment for multiple comparisons: Bonferroni

### Daily meal composition and variations during Ramadan among participating pupils in junior high schools

We assessed the number of dishes taken at meal times as well as the main dishes taken at meal times and their variation during Ramadan. Majority of pupils (98.3%) had one main dish at breakfast before the start of Ramadan but more dishes at dawn (breakfast) during Ramadan. For instance, while only 2 % of the pupils consumed two or more dishes at breakfast before Ramadan, more than four in ten pupils (46%) had two or more dishes at dawn two weeks into fasting which increased to nearly 50% during the last week of fasting and reduced to baseline value (2%) a month after Ramadan. Before Ramadan, pupils would have tea with bread (37%) or porridge with bread/roasted groundnut/beancake (36%) for breakfast. During Ramadan however, pupils would have tea with bread (62% and 57% for midline and endline respectively) as well as rice with stew (20% and 27% for midline and endline respectively) or *tuo zaafi* with soup (30% and 26% for midline and endline respectively). A month after Ramadan the pupils had returned to taking breakfast as either tea with bread (45.4%) or porridge with bread/roasted groundnut/beancake (33%) (Table 4).

**Table 4:** Changes in breakfast/dawn and lunch meals in Ramadan among participating pupils in JHS

	Baseline n/N (%)	Midline n/N (%)	Endline n/N (%)	Post Ramadan n/N (%)
Dish variety score of breakfast/dawn meals, mean ± SD	1.0 ± 0.13	1.5 ± 0.51	1.5 ± 0.52	1.0 ± 0.14
Dish variety at breakfast/dawn				
One dish	341/347 (98.3)	196/361 (54.3)	181/357 (50.7)	352/359 (98.1)
Two or more dishes	6/347 (1.7)	165/361 (45.7)	176/357 (49.3)	7/359 (1.9)
Breakfast/dawn meals				
Tea/tea and bread	128/347 (36.9)	223/361 (61.8)	202/357 (56.6)	163/359 (45.4)
Porridge (with kose/groundnut/bread)	124/347 (35.7)	11/361 (3.0)	11/357 (3.1)	119/359 (33.1)
Rice and stew (plain rice/jollof/fried rice/riceballs)	20/347 (5.8)	71/361 (19.7)	97/357 (27.2)	20/359 (5.6)
Rice and beans with stew	20/347 (5.8)	31/361 (8.6)	43/357 (12.0)	14/359 (3.9)
Tuo zaafi with soup	5/347 (1.4)	107/361 (29.6)	92/357 (25.8)	8/359 (2.2)
Banku with soup	2/347 (0.6)	20/361 (3.8)	16/357 (4.5)	2/359 (0.6)
Cocoa drink with bread	25/347 (7.2)	27/361 (7.5)	18/357 (5.0)	18/359 (5.0)
Fufu with soup	0/347 (0.0)	7/361 (1.9)	2/357 (0.6)	0/359 (0)
Fruits	0/347 (0.0)	2/361 (0.6)	1/357 (0.3)	0/359 (0)
Other foods	26/347 (7.5)	30/361 (8.3)	19/357 (5.3)	20/359 (5.6)
Dish variety score of lunch meals, mean ± SD	1.0 ± 0.15	1.2 ± 0.38	1.1 ± 0.29	1.01 ± 0.11
Dish variety at lunch				
One dish	326/334 (97.6)	11/13 (84.6)	11/12 (92)	334/338 (98.8)
Two or more dishes	8/334 (2.4)	2/13 (15.4)	1/12 (8)	4/338 (1.2)
Lunch meals				
Rice and stew (plain rice/jollof/fried rice/riceballs)	108/334 (32.3)	6/13 (46.2)	3/12 (25)	92/338 (27.2)
Rice and beans with stew	71/334 (21.3)	0/13 (0)	3/12 (25)	103/338 (30.5)
Tuo zaafi with soup	69/334 (20.7)	2/13 (15.4)	5/12 (42)	58/338 (17.2)
Banku with soup	16/334 (4.8)	1/13 (7.7)	0/12 (0)	12/338 (3.6)
Kenkey with stew	11/334 (3.3)	0/13 (0)	0/12 (0)	10/338 (3.0)
Yam meal (Wasawasa)	14/334 (4.2)	0/13 (0)	0/12 (0)	9/338 (2.7)
Fufu with soup (pounded yams and/cassava)	4/334 (1.2)	0/13 (0)	0/12 (0)	0/338 (0)
Fruits	1/334 (0.3)	0/13 (0)	0/12 (0)	0/338 (0)
Other foods	46/334 (13.8)	4/13 (30.8)	1/12 (8.3)	56/338 (16.6)

Foods usually taken before dinner which were used to break the fast during fasting doubled with Ramadan. For example, while majority (92% and 91%, respectively for baseline and post Ramadan) of the pupils had one dish outside Ramadan, they would have two or more dishes (68.4% and 71%, respectively for midline and endline) during Ramadan. Before Ramadan, pupils would have *tuo zaafi* with soup (23%) or mangoes (14.3%) or mashed kenkey (14.3%) before dinner time. Pupils however, had dates (60% and 61%, respectively for midline and endline) and mashed kenkey (54% and 56%, respectively for midline and endline) at *Iftar* (period to “breakfast”) during Ramadan. Fasting also came with a marked increase in the intake of watermelons and mangoes. One month after Ramadan, the pupils had one of the following; mangoes (24%) or watermelons (24%) or *tuo zaafi* with soup (18%) before dinner time.

The number of dishes taken at dinner remained unchanged during Ramadan at one dish. There were however, marked changes in the type of dishes taken. For example, while *tuo zaafi* was taken by the majority of the pupils (64% at baseline and post Ramadan) outside Ramadan, they had either rice with stew (36% and 35%, respectively for midline and endline) or *tuo zaafi* with soup (36% and 34%, respectively for midline and endline) for dinner during Ramadan (Table 5).

**Table 5:** Changes in foods taken before dinner/foods for breaking fast and dinner meals in Ramadan among participating pupils in JHS

	Baseline n/N (%)	Midline n/N (%)	Endline n/N (%)	Post Ramadan n/N (%)
Dish variety score of taken before dinner meals/ foods for breaking fast, mean ± SD	1.1 ± 0.3	2.1 ± 1.0	2.1 ± 0.9	1.1 ± 0.3
Dish variety of foods taken before dinner/foods for breaking fast				
One dish	103/112 (92.0)	99/312 (31.6)	98/339 (28.9)	96/106 (90.6)
Two or more dishes	9/112 (8.0)	214/312 (68.4)	241 (71.1)	10/106 (9.4)
Before dinner meals/ foods at *Iftar*				
Dates	0/112 (0.0)	187/312 (59.9)	209/339 (61.4)	0/106 (0.0)
Watermelon	1/112 (0.9)	61/312 (19.6)	97/339 (28.6)	25/106 (23.6)
Mango	16/112 (14.3)	62/312 (19.9)	64/339 (18.9)	0/106 (23.6)
Banana	3/112 (2.7)	19/312 (6.1)	15/339 (4.4)	1/106 (0.9)
Orange	1/112 (0.9)	12/312 (3.8)	6/339 (1.6)	1/106 (0.9)
Other fruits	3/112 (2.7)	6/312 (1.9)	6/339 (1.6)	1/106 (0.9)
Mashed kenkey	16/112 (14.3)	169/312 (54.2)	188/339 (55.5)	11/106 (10.4)
Porridge	7/112 (6.2)	58/312 (18.6)	59/339 (17.4)	7/106 (6.6)
Tea	1/112 (0.9)	11/312 (3.5)	6/339 (1.8)	3/106 (2.8)
Roselle drink (*Sobolo*)	13/112 (11.6)	6/312 (1.9)	9/339 (2.7)	7/106 (6.6)
*Tuo zaafi*/ Rice and stew	26/112 (23.2)	6/312 (1.9)	5/339 (1.5)	19/106 (17.9)
Other foods	36/112 (32.1)	49/312 (15.7)	30/339 (8.8)	36/106 (34.0)
Dish variety score of dinner meals, mean ± SD	1.0 ± 0.0	1.1 ± 0.4	1.1 ± 0.3	1.0 ± 0.1
Dish variety at dinner				
One dish	340/340 (100)	317/349 (90.8)	313/352 (88.9)	330/336 (98.2)
Two or more dishes	0/340 (0.0)	32/349 (9.2)	39/352 (11.1)	6/336 (1.8)
Dinner meals				
Rice and stew	57/340 (16.8)	128/349 (36.7)	123/352 (34.9)	47/336 (14.0)
Rice and beans	17/340 (5.0)	48/349 (13.8)	60/352 (17.0)	27/336 (8.0)
*Tuo zaafi* and soup	216/340 (63.5)	125/349 (35.8)	121/352 (34.4)	216/336 (64.3)
*Banku* and soup/stew	16/340 (4.7)	27/349 (7.7)	28/352 (8.0)	23/336 (6.8)
*Fufu* with soup	0/340 (0.0)	4/349 (1.1)	4/352 (1.1)	4/336 (1.2)
Fruits	0/340 (0.0)	5/349 (1.4)	3/352 (0.9)	0/336 (0.0)
Other foods	27/340 (7.9)	45/349 (12.9)	41/352 (11.6)	24/336 (7.1)
Foods after dinner				
Fruits	31/61 (50.8)	29/78 (37.2)	11/45 (24.4)	10/41 (24.4)

### Patterns of food groups and local dish consumption among participating pupils in junior high schools during Ramadan

**Fig. 1:**
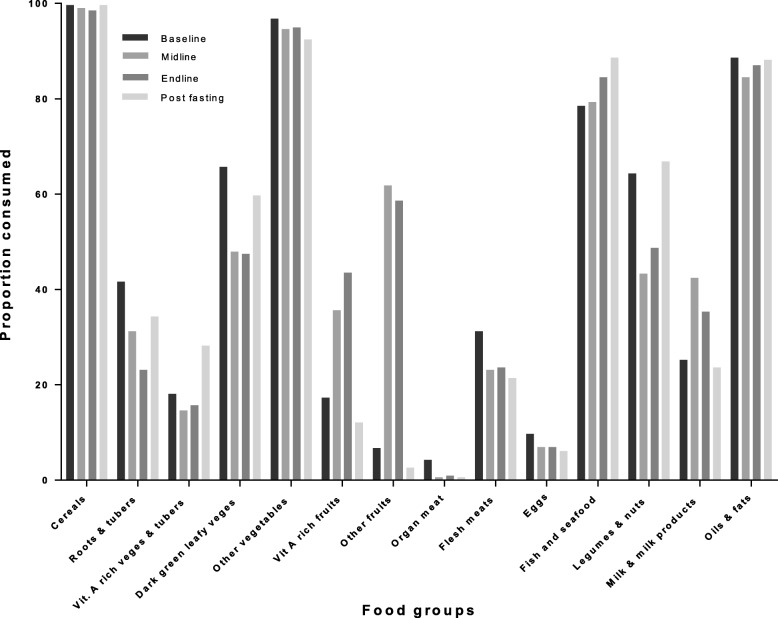
Proportion of food group consumption among participating pupils in JHS

The consumption from various food groups varied greatly in some food groups while others hardly changed during Ramadan. Consumption from cereals, other vegetables and fats and oils groups remained high without much changes with Ramadan. There was an increase in the consumption of vitamin A- rich fruits, other fruits, and milk and milk products during Ramadan. Fasting however, came with a reduction in consumption of foods from roots and tubers, legumes and nuts, vitamin A-rich vegetables and tubers and dark green leafy vegetables (Fig. 1).

**Fig. 2:**
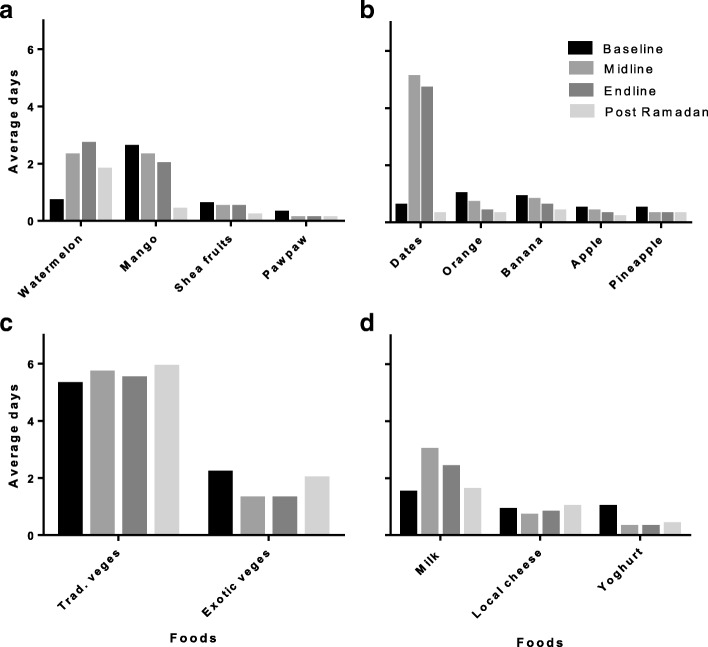
Patterns of consumption of vitamin A rich fruits (**a**), other fruits (**b**), vegetables (**c**) and dairy products (**d**) among participating pupils in JHS

Consumption patterns of vitamin A-rich fruits (watermelon, mango and shea fruit) generally increased during fasting except pawpaw which decreased during Ramadan. Intake of watermelons increased from about a day’s frequency to nearly three days a week during Ramadan. Mango intake was high, averaging nearly three days in a week before Ramadan and reduced slightly during Ramadan and further to a day’s frequency a month after Ramadan (Fig. 2a). Other fruits including pineapples, apples, bananas and oranges remained below a two day consumption frequency and almost unchanged with Ramadan. However, consumption of dates increased from below a two day frequency to nearly every day during Ramadan (Fig. 2b). Consumption of local vegetables (bra leaves (*Hibiscus sabdariffa*), *ayoyo* leaves (*Corchorus olitorius*), *aleefu* (*Amarantus sp*.), tomato, red hot pepper, onions, baobab leaves (dry), okro (fresh fruits, fruit powder)) remained high and almost unchanged while exotic vegetables (cabbage, lettuce, carrots, cucumber, green pepper) consumption fell slightly during Ramadan (Fig. 2c). Milk consumption increased from below two days in a week to above two days during Ramadan. Consumption frequency of other dairy foods such as yoghurt and local cheese remained below two days with no marked changes during Ramadan (Fig. 2d).

**Fig. 3:**
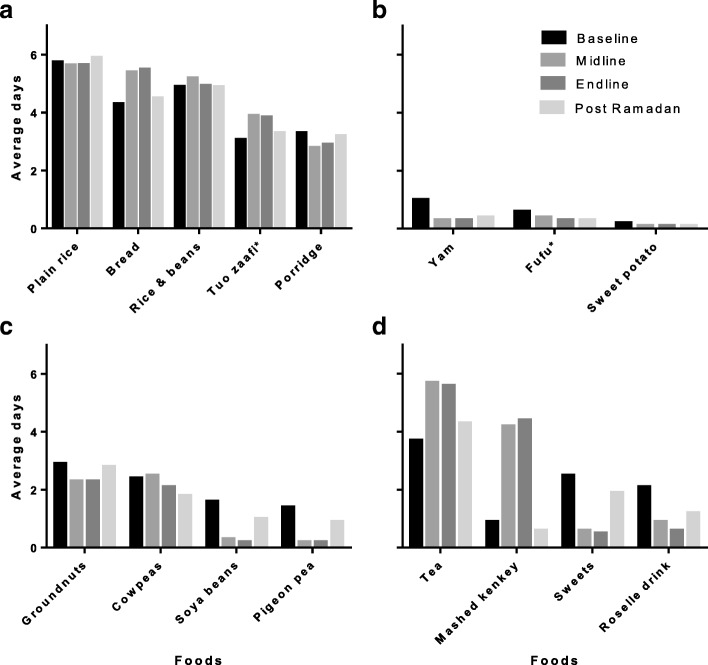
Patterns of consumption of cereal based foods (**a**), tuber based foods (**b**), legumes (**c**) and sugar sweetened beverages and sweets (**d**). *Tuo zaafi: made from maize/millet flour. *Fufu: Made from pounded yams and/or cassava

Commonly consumed cereal based staples maintained a daily consumption pattern (Fig. 3a). Such staples as *tuo zaafi*, plain rice, rice and beans, bread and porridge remained highly consumed, taken almost every day and unchanged during Ramadan. Tuber based foods were however, less frequently consumed before Ramadan and further reduced during Ramadan. For example, the consumption of *fufu* (made from pounded cassava and/or yams), boiled yams and sweet potatoes decreased during fasting and began to rise one month after fasting (Fig. 3b). There was a reduction in the consumption of leguminous foods such as soya beans (usually eaten as soya kebab), pigeon pea, groundnuts, and cowpea with varying patterns. While frequency of groundnut intake fell to below three days in a week during Ramadan and rose to baseline frequency a month after Ramadan, intake of cowpeas remained similar to baseline frequency of a little above three days at midline assessment and reduced thereafter at endline and post Ramadan assessments. Pattern of soya bean and pigeon pea consumption were similar with a little above two days weekly consumption frequency at baseline assessment which reduced steeply to below a day’s frequency during Ramadan and began to rise to baseline frequency at post Ramadan assessment (Fig. 3c). Tea intake increased from a little below four days to almost six days in a week during Ramadan. Mashed *kenkey* (made from fermented corn dough with added sugar) consumption increased markedly from about once a week to nearly four times during Ramadan and reduced thereafter. Further, fasting came with a marked decrease in intake of sweets (toffees, gums). Before Ramadan, pupils would have sweets for about three times in a week but had sweets for less than a day in a week during Ramadan (Fig. 3d).

Meat (beef, mutton and chevon) consumption patterns remained relatively the same averaging three days in a week while poultry (chicken, guinea fowl, turkey) intake experienced a marginal decrease during Ramadan. There was nearly a one day increase in consumption of fish and sea foods during Ramadan which increased further after fasting. Intake of fats and oils remained high and relatively unchanged during the first half of Ramadan but reduced in the second half of Ramadan. There was lower consumption frequency of chocolates, fried rice (largely a fast food), energy drinks, coffee, yoghurt, soft drinks, and packed fruit drinks across assessment points which further reduced during Ramadan or remained unchanged (soft drinks and packed fruit juice) with frequencies of once or less in a week (See Additional file 1).

### Changes in dietary diversity among participating pupils in junior high schools during Ramadan

Using a repeated measure ANOVA with a Greenhouse-Geisser correction, it was found that the differences in mean dietary diversity were statistically significant across the assessment stages (F (2.933, 1070.573) = 7.152, *p* < 0.001). There was a marginal increase in mean dietary diversity score from baseline (6.5 ± 1.6), midline (6.6 ± 1.8) through to the endline (6.7 ± 1.7) and decreased thereafter at post Ramadan (6.2 ± 1.4). Post hoc test using Bonferroni correction revealed a significant decrease in mean dietary diversity scores between midline and post Ramadan assessments (*p* < 0.001) and between endline and post Ramadan assessments (*p* < 0.001). There was however, no statistically significant mean difference between baseline and midline; baseline and endline; and baseline and post Ramadan assessments (*p* > 0.05) (Table 6). Differences in mean dietary diversity scores between males and females across assessment points were investigated using t- test. Females generally had higher dietary diversity scores outside Ramadan (baseline and post Ramadan) but lower during Ramadan (midline and endline), while the males had lower dietary diversity scores outside Ramadan but higher during Ramadan. Mean dietary diversity scores however, did not differ significantly between males and females at baseline (6.4 vs 6.5, *p* > 0.05), endline (6.7 vs 6.6, p > 0.05) and post Ramadan (6.2 vs 6.3, *p* > 0.05) but at midline (6.9 vs 6.4, *p* = 0.002) (Results not shown).

**Table 6:** Pairwise comparisons of changes in dietary diversity among participating pupils in JHS (Repeated measures ANOVA)

(I) Dietary diversity (mean ± SD)	(J) dietary diversity (mean ± SD)	Mean Difference (I-J)	Std. Error	Sig.^b^	95% Confidence Interval for Difference^b^	
					Lower Bound	Upper Bound
Baseline diversity (6.5 ± 1.6)	Midline diversity (6.6 ± 1.8)	−0.175	0.113	0.734	−0.474	0.125
	Endline diversity (6.7 ± 1.7)	−0.210	0.116	0.418	−0.517	0.096
	Post Ramadan diversity (6.2 ± 1.4)	0.235	0.102	0.127	−0.034	0.504
Midline diversity (6.6 ± 1.8)	Endline diversity (6.7 ± 1.7)	− 0.036	0.108	1.000	− 0.322	0.251
	Post Ramadan diversity (6.2 ± 1.4)	0.410^a^	0.105	0.001	0.132	0.688
Endline diversity (6.7 ± 1.7)	Post Ramadan diversity (6.2 ± 1.4)	0.445^a^	0.102	< 0.001	0.174	0.717

Based on estimated marginal means, ^a^. The mean difference is significant at the .05 level, ^b^Adjustment for multiple comparisons: Bonferroni

### Weight changes among participating pupils in junior high schools during Ramadan

Our data showed statistically significant weight changes across the assessment points in a repeated measure ANOVA using Greenhouse-Geisser correction (F (2.656, 958.95) = 304.90, *p* < 0.001). Fasting explained about 46% of weight changes (Partial Eta Squared = 0.458) in pupils during Ramadan. There were significant weight loses from baseline through to midline and endline assessments (*p* < 0.001). For example, pupils lost an average of 1.1 kg of weight between baseline and midline assessments and 1.5 kg (95% CI: -1.1 kg to − 1.6 kg) by endline assessment. Overall weight loss during Ramadan was significantly higher among females (− 1.7 kg, 95% CI: -1.6 kg to − 1.9 kg) than males (− 1.2 kg, 95% CI: -1.0 kg to − 1.3 kg) (p < 0.001). Weight loss did not differ by age of pupil (*p* > 0.05) (Results not shown). Pupils had however, recovered their lost weight one month after Ramadan, evident in the lack of significant difference (*p* = 0.233) between mean baseline weight (51.1 ± 8.9) and post fasting weight (51.0 ± 8.8) (Table 7). In a t-test, it was revealed that mean weight of pupils did not differ by sex across all assessment points (p > 0.05) (Results not shown).

**Table 7:** Pairwise comparisons of changes in body weight among participating pupils in JHS (Repeated measures ANOVA)

(I) Weight changes (mean ± SD)	(J) Weight changes (mean ± SD)	Mean Difference (I-J)	Std. Error	Sig.^b^	95% Confidence Interval for Difference^b^	
					Lower Bound	Upper Bound
Baseline weight (51.1 ± 8.9)	Midline weight (50.0 ± 8.9)	1.085^a^	0.054	< 0.001	0.942	1.229
	Endline weight (49.6 ± 8.8)	1.452^a^	0.057	< 0.001	1.301	1.602
	Post Ramadan weight (51.0 ± 8.8)	0.140	0.068	0.233	−0.039	0.319
Midline weight (50.00 ± 8.87)	Endline weight (49.6 ± 8.8)	0.366^a^	0.045	< 0.001	0.248	0.485
	Post Ramadan weight (51.0 ± 8.8)	−0.945^a^	0.063	< 0.001	−1.113	−0.778
Endline weight (49.63 ± 8.81)	Post Ramadan weight (51.0 ± 8.8)	−1.312^a^	0.056	< 0.001	− 1.460	− 1.163

Based on estimated marginal means, ^a^. The mean difference is significant at the .05 level, ^b^Adjustment for multiple comparisons: Bonferroni

## Discussion

This study sought to describe the food patterns, dietary diversity and weight changes among Ghanaian adolescents during Ramadan. The main findings were that daily meal frequency reduced significantly during Ramadan. There were marked changes in the number and types of dishes taken at meal times during Ramadan. In addition, dietary diversity increased significantly during Ramadan. Consumption of some food groups varied greatly while others hardly changed during Ramadan. Adolescent food intake during Ramadan was largely characterised by increases in fruit and vegetable consumption, dish variety, dietary diversity, reduced intakes of fast foods (such as fried rice), energy and soft drink, sweets, fats and oils and high intake of cereal based foods. Further, there was statistically significant body weight loss of 1.5 kg over the Ramadan period.

The significant reduction in the number of eating moments during Ramadan is not surprising because the whole daytime is typically spent without food during Ramadan. Daytime eating moments are therefore essentially lost. Significant decrease in daily meal frequency during Ramadan has been reported earlier [26].

The increased dish variety at dawn during Ramadan could be explained by the temptation to combine usual breakfast and lunch to provide sustenance for the day’s fast. Pupils therefore, took a lighter meal (tea with/out bread) similar to their usual breakfast outside Ramadan in addition to a heavy meal (rice with stew or *tuo zaafi* with soup) similar to what they would normally have for lunch. Energy and nutrient intake from the increased dishes at dawn is however, not expected to be similar to energy and nutrient intakes from a combined usual breakfast and lunch outside Ramadan as increasing dishes at meal times could mean reduced portion sizes for both dishes [27]. In addition, appetite is likely to be suppressed at dawn due to the nocturnal rise in leptin levels among non-fasting people [28] and those fasting in Ramadan [29]. The increase in dish variety during *Iftar* may be due to the usual practice of taking fruits at *Iftar*.

The significant increase in dietary diversity during Ramadan could be due to increase in specific food groups such as other fruits, vitamin A rich fruits, and milk and milk products. The increase in fruits consumption were largely due to the marked increase in intake of dates, mangoes and watermelons normally taken at *Iftar* during Ramadan. The increase in milk and milk products intake is understandable as there was marked increase in tea intake which is served with milk. This increase could also be due to the increase in mashed *kenkey* which could be served with milk. The decrease in consumption from the legumes and nuts, and dark green leafy vegetables food groups may mean that these foods are normally taken at school as lunch. Rice and cowpeas (*Waaky*e), and pigeon peas which are major legume based foods are normally served with assorted vegetables including cabbage, lettuce and carrots at school for lunch [30]. The marginally higher dietary diversity scores during Ramadan among males compared to females could be explained by the higher tendency of males to have other foods as gifts at *Iftar* usually provided at the Mosque in addition to family-provided meals. In addition, girls are likely to be home helping prepare meals while boys are outside grabbing whatever may be available.

Generally, we find adolescent food intakes during Ramadan to follow a relatively healthy diet pattern. Dietary intake was characterised by increase in fruit and vegetable consumption, increased dish variety, increased dietary diversity, reduced intake of fast foods (such as fried rice), reduced energy drink and soft drink intake, reduced intake of sweets, reduced fats and oils intake and high intake of cereal based foods. Healthy diet recommendations for adolescents stress the importance of fruits and vegetables, whole grains, fish, low fat dairy and lean meat intake [31, 32]. Increased dietary diversity has also been linked to specific nutrient adequacy [20] and represents an appropriate approach to measuring nutrient adequacy among adolescents [17] including those in resource poor settings [18]. This notwithstanding, nutrition education for adolescents during Ramadan should encourage reduction of table sugar in sugar sweetened beverages such as tea and mashed *kenkey* which are increased considerably during Ramadan.

Findings have been mixed regarding weight changes during Ramadan. Self-reported weight gain has been reported among a cohort of Saudi families [33], while other studies report unchanged weight during Ramadan [26, 34]. Conflicting results regarding weight changes among people who fast during Ramadan in different settings could be due to differences in dietary behaviours in different cultures, differences in fast duration and variations in climatic conditions. The significant weight loss among adolescents in present study is consistent with previous studies where a little above 1 kg of body weight was lost during Ramadan and regained shortly afterwards [35–37]. A recent study suggested that weight changes during Ramadan are more likely the result of differences in food intake [38]. Reduced nutrient intakes in adolescents have been reported earlier [13]. Smaller portion sizes may be consumed at dinner due to high intake of water following a prolonged thirst, increased intake of less energy dense fluids and fruits at *Iftar* as well as the shorter time between *Iftar* and dinner meals. In addition, daytime food quantities may be smaller due to lower appetite at dawn as well as a non-consumption of lunch meals and other lunch snacks during Ramadan. The significantly high loss in body weight among female adolescents is contrary to earlier findings reported among Malaysian adolescents [13] and among adults elsewhere [35]. Differences in activity level and food intake among males and females could account for the disparity in weight loss. Energy intake was more likely to be higher among males as they had multiple eating points – at home and as gifts at the Mosque during Ramadan. Males are also more likely to spend more time sleeping after school than females who are usually active at home assisting with food preparations and other household chores. The significant weight losses among adolescent females may have some important implications. Weight losses during Ramadan therefore mean a trade-off of nutrients for weight regain at the expense of growth and preparation towards future reproductive functions. Yearly weight losses among female adolescents over the period of adolescence could have cumulative important implications on their nutrition and health. It seems reasonable therefore, for female adolescents preparing for Ramadan to receive nutrition education on dietary practices essential in maintaining nutrient stores and optimal weight.

The strengths of this study include the use of a longitudinal design and a relatively large sample. Our study is however, constrained by the use of qualitative dietary assessment methods (food frequency questionnaire and 24-h dietary recall) which do not allow us to make quantitative estimates of food intake. It is important to note however, that the food frequency questionnaire is shown to be a useful tool in assessing eating habits of older children and adolescents [39]. It is also a practical tool for assessing usual intake of subjects, is relatively easy to complete and useful in prospective studies involving large numbers of subjects [40, 41]. Obviously quantitative diet assessments are useful, but may not always be feasible (due to high cost, logistics and poor adherence by subjects) especially in a prospective study involving large numbers of subjects [40]. Even though the 24-h recall method has an inherent limitation of relying on respondent’s memory and respondents might not always be entirely truthful, the method has been thought to provide data which represent the population due to lower respondent burden compared with the diet record method [40].

Further, compensatory feeding is more likely in fasting conditions, but present study did not measure this behaviour. However, the increased number of dishes at major eating moments during Ramadan is suggestive of the behaviour. If more foods and fluids were taken at dawn to compensate for the absence of lunch meals and the day’s thirst, then pupils were more likely to be heavier around the time (morning) of weight measurement during Ramadan than they would be outside Ramadan where breakfast meals could even be skipped with no much worries as pupils still have the chance to have lunch meals during the day. This could have led to an under estimation of weight loss. In spite of these limitations, the present study has shed much light on the food patterns, dietary diversity and weight changes among adolescents who fast during Ramadan in Ghana.

## Conclusion

In this prospective cohort study among schooling Ghanaian adolescents who fast during Ramadan, fasting was characterised by marked changes in usual food patterns, increased dietary diversity and significant body weight loss.

## Additional file

Additional file 1: Patterns of other foods consumed by participating pupils during Ramadan (7 day frequency). (DOCX 15 kb)

## Abbreviations

ANOVA: Analysis of variance
DDS: Dietary diversity score
JHS: Junior High School

## References

[1] Erol A, Baylan G, Yazici F (2008). Do Ramadan fasting restrictions alter eating behaviours?. Eur Eat Disord Rev.

[2] Trepanowski JF, Bloomer RJ (2010). The impact of religious fasting on human health. Nutr J.

[3] Sakr AH (1975). Fasting in Islam. J Am Diet Assoc.

[4] Farooq A, Herrera CP, Almudahka F, Mansour R (2015). A prospective study of the physiological and neurobehavioral effects of ramadan fasting in preteen and teenage boys. J Acad Nutr Diet.

[5] Fenneni MA, Latiri I, Aloui A, Rouatbi S, Saafi MA, Bougmiza I, Chamari K, Saad HB. Effects of Ramadan on physical capacities of North African boys fasting for the first time. *Libyan J Med*. 2014;9 10.3402/ljm.v3409.25391. 10.3402/ljm.v9.25391 PMC4176671 25261691

[6] Ramadan J (2002). Does fasting during Ramadan alter body composition, blood constituents and physical performance?. Med Princ Pract.

[7] Nematy M, Alinezhad-Namaghi M, Rashed MM, Mozhdehifard M, Sajjadi SS, Akhlaghi S, Sabery M, Mohajeri SAR, Shalaey N, Moohebati M (2012). Effects of Ramadan fasting on cardiovascular risk factors: a prospective observational study. Nutr J.

[8] Leiper J, Molla A (2003). Effects on health of fluid restriction during fasting in Ramadan. Eur J Clin Nutr.

[9] Akrami Mohajeri F, Ahmadi Z, Hassanshahi G, Akrami Mohajeri E, Ravari A, Ghalebi SR (2013). Dose Ramadan fasting affects inflammatory responses: evidences for modulatory roles of this unique nutritional status via chemokine network. Iranian J Basic Med Sci.

[10] Al-Hourani H, Atoum M (2007). Body composition, nutrient intake and physical activity patterns in young women during Ramadan. Singap Med J.

[11] Azizi F (2010). Islamic fasting and health. Ann Nutr Metab.

[12] Toda M, Morimoto K (2000). Effects of Ramadan fasting on the health of Muslims. Nihon eiseigaku zasshi Japanese j hygiene.

[13] Poh B, Zawiah H, Ismail M, Henry C (1996). Changes in body weight, dietary intake and activity pattern of adolescents during Ramadan. Malays J Nutr.

[14] Akgül S, Derman O, Kanbur NÖ (2014). Fasting during ramadan: a religious factor as a possible trigger or exacerbator for eating disorders in adolescents. Int J Eat Disord.

[15] World Health Organization (2014). Health for the world's adolescents: a second chance in the second decade: summary.

[16] Croll JK, Neumark-Sztainer D, Story M (2001). Healthy eating: what does it mean to adolescents?. J Nutr Educ.

[17] Mirmiran P, Azadbakht L, Esmaillzadeh A, Azizi F (2004). Dietary diversity score in adolescents-a good indicator of the nutritional adequacy of diets: Tehran lipid and glucose study. Asia Pac J Clin Nutr.

[18] Steyn N, Nel J, Nantel G, Kennedy G, Labadarios D (2006). Food variety and dietary diversity scores in children: are they good indicators of dietary adequacy?. Public Health Nutr.

[19] Arimond M, Wiesmann D, Becquey E, Carriquiry A, Daniels MC, Deitchler M, Fanou-Fogny N, Joseph ML, Kennedy G, Martin-Prevel Y (2010). Simple food group diversity indicators predict micronutrient adequacy of women’s diets in 5 diverse, resource-poor settings. J Nutr.

[20] Mirmiran P, Azadbakht L, Azizi F (2006). Dietary diversity within food groups: an indicator of specific nutrient adequacy in Tehranian women. J Am Coll Nutr.

[21] Ghana Statistical Service. 2010 Population and Housing Census summary report of final results. Accra: Ghana Statistical Service; 2012.

[22] Ghana Statistical Service. 2010 Population and Housing Census Distric analytical report Tamale Metropolis. Accra: Ghana Statistical Service; 2014.

[23] Kennedy G, Ballard T, Dop MC (2011). Guidelines for measuring household and individual dietary diversity: food and agriculture Organization of the United Nations.

[24] Abubakari A, Jahn A (2016). Maternal dietary patterns and practices and birth weight in northern Ghana. PLoS One.

[25] WHO (1995). Physical status: The use of and interpretation of anthropometry, Report of a WHO Expert Committee.

[26] Frost G, Pirani S (1987). Meal frequency and nutritional intake during Ramadan: a pilot study. Human nutrition Applied nutrition.

[27] Vermeer WM, Steenhuis IH, Leeuwis FH, Heymans MW, Seidell JC (2011). Small portion sizes in worksite cafeterias: do they help consumers to reduce their food intake?. Int J Obes.

[28] Sinha MK, Ohannesian JP, Heiman ML, Kriauciunas A, Stephens TW, Magosin S, Marco C, Caro JF (1996). Nocturnal rise of leptin in lean, obese, and non-insulin-dependent diabetes mellitus subjects. J Clin Invest.

[29] Ajabnoor GM, Bahijri S, Borai A, Abdulkhaliq AA, Al-Aama JY, Chrousos GP (2014). Health impact of fasting in Saudi Arabia during Ramadan: association with disturbed circadian rhythm and metabolic and sleeping patterns. PLoS One.

[30] Abizari A-R, Buxton C, Kwara L, Mensah-Homiah J, Armar-Klemesu M, Brouwer ID (2014). School feeding contributes to micronutrient adequacy of Ghanaian schoolchildren. Br J Nutr.

[31] Gidding SS, Dennison BA, Birch LL, Daniels SR, Gilman MW, Lichtenstein AH, Rattay KT, Steinberger J, Stettler N, Van Horn L (2005). Dietary recommendations for children and adolescents: a guide for practitioners: consensus statement from the American Heart Association. Circulation.

[32] Hussein L, Gouda M, Fouad M, Labib E, Bassyouni R, Mohammad M (2014). Dietary intervention with yoghurt, Synbiotic yogurt or traditional fermented Sobya: bio-potency among male adolescents using five bio-markers of relevance to colonic metabolic activities. Food Nutr Sci.

[33] Bakhotmah BA (2011). The puzzle of self-reported weight gain in a month of fasting (Ramadan) among a cohort of Saudi families in Jeddah, western Saudi Arabia. Nutr J.

[34] Lamine F, Bouguerra R, Jabrane J, Marrakchi Z, Ben MR, Ben CS, Gaigi S (2006). Food intake and high density lipoprotein cholesterol levels changes during ramadan fasting in healthy young subjects. La Tunisie Médicale.

[35] Sadeghirad B, Motaghipisheh S, Kolahdooz F, Zahedi MJ, Haghdoost AA (2012). Islamic fasting and weight loss: a systematic review and meta-analysis. Public Health Nutr.

[36] Hajek P, Myers K, Dhanji A-R, West O, McRobbie H (2011). Weight change during and after Ramadan fasting. J Pub Health.

[37] Syam AF, Sobur CS, Abdullah M, Makmun D (2016). Ramadan fasting decreases body fat but not protein mass. Int j endocrinol metabol.

[38] Lessan N, Saadane I, Alkaf B, Hambly C, Buckley AJ, Finer N, Speakman JR, Barakat MT (2018). The effects of Ramadan fasting on activity and energy expenditure. Am J Clin Nutr.

[39] Rockett HR, Wolf AM, Colditz GA (1995). Development and reproducibility of a food frequency questionnaire to assess diets of older children and adolescents. J Acad Nutr Diet.

[40] Thompson FE, Subar AF. Dietary assessment methodology. In: *Nutrition in the Prevention and Treatment of Disease (Third Edition)*. New York: Elsevier; 2013. p. 5–46.

[41] Willett W (1998). Nutritional epidemiology.

