# Supplementary material for: Ramadan fasting alters food patterns, dietary diversity and body weight among Ghanaian adolescents
Source: Nutr J. 2018 Aug 11;17:75. doi: 10.1186/s12937-018-0386-2 (PMC6086999; doi:10.1186/s12937-018-0386-2)
Supplement: Supplementary file 1 — Patterns of other foods consumed by participating pupils during Ramadan (7 day frequency). (DOCX 15 kb) [file 12937_2018_386_MOESM1_ESM.docx]

## Additional file 1

| **Study Arm** | **Soft drinks** | **Poha** | **Fats & oils** | **Zimkuom** | **Fula** | **Jollof** | **Meat** | **Fish** | **Poultry** | **Eggs** | **Chocolate** | **Fried rice** | **Biscuit** | **Energy drinks** | **Fan milk** | **Fanchoco** |
| --- | --- | --- | --- | --- | --- | --- | --- | --- | --- | --- | --- | --- | --- | --- | --- | --- |
| **Baseline** | 1.0 | 1.3 | 5.0 | 1.0 | 0.2 | 1.2 | 2.4 | 3.0 | 0.7 | 1.2 | 0.7 | 0.5 | 2.2 | 0.7 | 0.4 | 0.5 |
| **Midline** | 1.4 | 0.6 | 5.0 | 0.7 | 0.1 | 1.3 | 2.3 | 3.8 | 0.4 | 1.0 | 0.2 | 0.3 | 0.7 | 0.3 | 0.2 | 0.3 |
| **Endline** | 0.6 | 0.3 | 4.9 | 0.7 | 0.04 | 1.3 | 2.4 | 3.9 | 0.4 | 0.9 | 0.3 | 0.2 | 0.8 | 0.3 | 0.2 | 0.2 |
| **Post fast** | 0.6 | 0.4 | 4.7 | 0.3 | 0.1 | 1.0 | 2.1 | 4.4 | 0.5 | 0.8 | 0.4 | 0.4 | 1.4 | 0.4 | 0.2 | 0.2 |

**1^a^ Patterns of other foods consumed during Ramadan (7 day frequency)**

**1^b^ Patterns of other foods consumed during Ramadan (7 day frequency)**

| **Study Arm** | **Kenkey** | **Plantain** | **coffee** | **Maasa** | **Yoghurt** | **Pear** | **Berries** | **Honey** | **Malt** | **Pepper bear** | **Fruit juice** | **Bambara beans** | **palmnut** | **Coconut** |
| --- | --- | --- | --- | --- | --- | --- | --- | --- | --- | --- | --- | --- | --- | --- |
| **Baseline** | 1.1 | 0.5 | 0.4 | 0.6 | 1.0 | 1.2 | 0.3 | 0.7 | 0.6 | 0.4 | 0.5 | 0.4 | 0.9 | 0.5 |
| **Midline** | 0.7 | 0.2 | 0.3 | 0.3 | 0.3 | 0.4 | 0.2 | 0.4 | 0.3 | 0.1 | 0.5 | 0.2 | 0.7 | 0.2 |
| **Endline** | 0.6 | 0.2 | 0.3 | 0.3 | 0.3 | 0.2 | 0.2 | 0.2 | 0.2 | 0.1 | 0.4 | 0.1 | 0.7 | 0.1 |
| **Post fast** | 0.9 | 0.3 | 0.2 | 0.4 | 0.4 | 0.1 | 0.1 | 0.3 | 0.3 | 0.1 | 0.2 | 0.2 | 0.5 | 0.2 |
